# Genetic associations between alcohol phenotypes and life satisfaction: a genomic structural equation modelling approach

**DOI:** 10.1038/s41598-023-40199-1

**Published:** 2023-08-18

**Authors:** Kaitlin E. Bountress, Shannon E. Cusack, Sage E. Hawn, Andrew Grotzinger, Daniel Bustamante, Robert M. Kirkpatrick, Howard J. Edenberg, Ananda B. Amstadter

**Affiliations:** 1 Virginia Institute for Psychiatric and Behavioral Genetics, Virginia Commonwealth University, 800 E. Leigh St. Biotech One Suite 101, Richmond, VA 23219 USA; 2 Department of Psychology, Old Dominion University, Norfolk, USA; 3 Institute for Behavior Genetics, Behavioral, Psychiatric, and Statistical Genetics, University of Colorado Boulder, Boulder, USA; 4 School of Medicine, Indiana University, Bloomington, IN USA

**Keywords:** Genomics, Genetics, Psychology

## Abstract

Alcohol use (i.e., quantity, frequency) and alcohol use disorder (AUD) are common, associated with adverse outcomes, and genetically-influenced. Genome-wide association studies (GWAS) identified genetic loci associated with both. AUD is positively genetically associated with psychopathology, while alcohol use (e.g., drinks per week) is negatively associated or NS related to psychopathology. We wanted to test if these genetic associations extended to life satisfaction, as there is an interest in understanding the associations between psychopathology-related traits and constructs that are not just the absence of psychopathology, but positive outcomes (e.g., well-being variables). Thus, we used Genomic Structural Equation Modeling (gSEM) to analyze summary-level genomic data (i.e., effects of genetic variants on constructs of interest) from large-scale GWAS of European ancestry individuals. Results suggest that the best-fitting model is a Bifactor Model, in which unique alcohol use, unique AUD, and common alcohol factors are extracted. The genetic correlation (*r*_*g*_) between life satisfaction-AUD specific factor was near zero, the *r*_*g*_ with the alcohol use specific factor was positive and significant, and the *r*_*g*_ with the common alcohol factor was negative and significant. Findings indicate that life satisfaction shares genetic etiology with typical alcohol use and life dissatisfaction shares genetic etiology with heavy alcohol use.

## Introduction

Alcohol use is common; 71.7% of individuals in the United States aged 15 years and older endorse drinking at least one drink in the past year^1^. Alcohol use in excess (e.g., binge drinking, i.e., 4 + drinks/occasion for women and 5 + for men, or heavy drinking, 8 + drinks/week for women and 15 + drinks/week for men)^2^ is associated with a number of deleterious outcomes including the development of alcohol use disorder (AUD; Ref.^3^). It is estimated that, in the United States, ~ 6% of individuals meet criteria for a past year AUD^4^, posing significant public health costs^5,6^. Given the notable prevalence of and consequences associated with excess alcohol use and AUD, there is a need to better understand their etiology in order to improve prevention and intervention efforts.

Behavioral genetic studies on AUD and alcohol use have estimated the twin-based heritability of AUD to be 0.49 for a review see Ref.^7^; 95% CI 0.43–0.53 and alcohol use (i.e., quantity and frequency of drinking) to be 0.43^8^; 95% CI 0.31–0.56. Recent molecular work on the genetic influences on AUD and alcohol use have also yielded modest SNP-based heritability (${h}_{SNP}^{2}$) estimates ranging from 5.6 to 13.0% for AUD^9^; h^2^ = 0.056, S.E. = 0.004^10^; h^2^ = 0.094, S.E. = 0.005, alcohol use^11^; h^2^ = 0.13, S.E. = 0.01 and problematic alcohol use, including summary statistics for AUD diagnosis, alcohol dependence (AD) diagnosis and scores on a measure of problems experienced related to one’s use (i.e., Alcohol Use Disorders Identification Test- Problems/AUDIT-P)^10^; h^2^ = 0.068, S.E. = 0.004. Genome-wide association studies (GWAS) have identified genome-wide significant SNPs associated with AUD and problematic alcohol use i.e., ADH1B and ADH1C genes; Refs.^9,10,12–15^ and with alcohol use in the general population e.g., Refs.^11,16,17^. Furthermore, Kranzler, et al.^9^ found a positive, significant genetic correlation between alcohol use, as assessed using the Alcohol Use Disorders Test- Consumption (AUDIT-C) questions on quantity and frequency of alcohol use, and AUD (r_g_ = 0.52). Mallard, et al.^18^ found evidence of a correlated two-factor structure (i.e., “consumption” and problems” [r_g_ = 0.80]) similarly using the AUDIT. Taken together, these findings highlight that the genetic influences on AUD and alcohol use are correlated but not at unity. Studies of alcohol use in population samples are often dominated by the majority of individuals who drink in moderation, which might explain part of the difference. In addition, recent work finds that, phenotypically, most of the variance in alcohol-related consequences is not driven by alcohol use^19^.

Although extant molecular studies provide a foundation for examining the etiologic underpinnings of alcohol use and AUD, both phenotypes are highly polygenic, indicating a genetic architecture comprised of thousands of causal variants^9,13^. High polygenicity then necessitates statistical genetic techniques that can be used to summarize the relationships among aggregate genetic risk for alcohol use, AUD, and relevant clinical correlates. Indeed, using linkage disequilibrium score regression LDSR; Ref.^20^, several groups have found different patterns of associations of alcohol use and AUD with other traits^9,10,21^.

Further, the genetic relationship between mental health phenotypes (i.e., PTSD, anxiety, and depression) and phenotypes indexing problematic alcohol use/symptoms of AUD versus non-pathological use differs. Our group found significant, positive genetic correlations between posttraumatic stress disorder (PTSD) and AUD-related phenotypes (e.g., AUDIT-P scores, maximum alcohol intake, AUD, and AD), but negative (significant and non-significant) associations between PTSD and alcohol use-related phenotypes (e.g., drinks per week, AUDIT-C; Ref.^22^. Another group found a near zero genetic correlation between PTSD and the AUDIT-C subscale score (which measures quantity and frequency of use, rather than problems related to drinking)^18^. LDSC analyses also demonstrated that problematic use (i.e., an as assessment of problems experienced due to alcohol use) and AD are positively correlated with both anxiety^23^ and depression symptoms^24^, but that non-pathological alcohol use phenotypes (e.g., quantity, frequency) are not correlated with anxiety^23^ and negatively correlated depression symptoms^24^.

While these studies have been useful in giving us a preliminary sense of varying genetic architecture underlying alcohol use and AUD via pairwise genetic associations among constructs, the techniques used do not allow researchers to examine associations while accounting for other factors/constructs. Since the genetic architectures of alcohol use and AUD are correlated but distinct, approaches estimating variance that is common to and unique from these two phenotypes may be useful. Genomic Structural Equation Modeling (gSEM; Ref.^25^) is a novel statistical genetic technique that builds upon LDSC to fit multivariate models of genetic associations, allowing researchers to identify the latent genetic factor structure of multiple phenotypes. This approach makes it possible to index genetic overlap among phenotypes, as well as variance that is unique to each trait (e.g., alcohol use quantity and frequency vs. AUD). Indeed, work by our group using gSEM found that the best fitting model was one that differentiated genetic factors for alcohol use and AUD relative to models with all alcohol-related indicators loading onto a single factor^26^. Further, this work demonstrated that the genetic correlation with PTSD for a common alcohol factor indexing shared genetic variance across alcohol indicates was null. This was in comparison to positive and negative associations between the PTSD-AUD factor and the PTSD-alcohol use factor, respectively.

One potential hypothesis for these findings is that those who drink to the point of disorder do so because they are unhappy or unsatisfied with something in their lives. In contrast, individuals who drink at more moderate levels may be consuming alcohol to celebrate happy occasions or because they feel happy or satisfied with their lives. Thus, one might hypothesize that the genetic correlation between life satisfaction and a unique AUD factor might be negative, and the genetic correlation between life satisfaction and a unique alcohol use factor might be positive. Most of the phenotypic work examining associations between alcohol phenotypes and other outcomes has focused on deficits and psychopathology, and this literature has largely neglected protective and/or positive factors that may be associated with alcohol outcomes. Work examining associations between alcohol and positive factors is critical to informing the etiology, prevention and intervention efforts in this area. Examining this question from a genetic perspective allows us to understand the genetic architecture of a range of alcohol phenotypes.

The limited phenotypic work on life satisfaction and alcohol phenotypes suggests that lower levels of satisfaction are associated with more alcohol problems; however, there generally seems to be no association between life satisfaction and alcohol use^27,28^. Given this discrepant pattern of findings in this rather small literature, and the fact that life satisfaction itself is moderately heritable (h^2^: 38; Ref.^29^), it would be useful to know if the genetic correlations, explored in a gSEM framework, between life satisfaction and alcohol phenotypes, follows the pattern outlined above such that on a genetic level, alcohol use and alcohol related problems/AUD show varying results with regard to their association with life satisfaction. If this pattern of findings holds, our work provides additional evidence that the genetic underpinnings of alcohol use vary, at least in part, from those underlying problematic use and/or disorder.

The primary aim of the present paper was to examine the genetic architectures of alcohol use, AUD, and life satisfaction. We sought to examine if the direction of effect between alcohol use and life satisfaction, AUD and life satisfaction, and a combined alcohol factor and life satisfaction were discrepant. We first used gSEM to test whether two or three factor solutions fit better than the one factor model. We then tested the hypothesis that the genetic associations between and alcohol use phenotypes would be significant and positive, and between life satisfaction and AUD would be significant and negative. We also hypothesized that a model separating what is common to all the alcohol items while leaving what is unique to alcohol use and AUD would show even better fit, with the genetic correlations between life satisfaction indices and the common alcohol factor being near zero, life satisfaction and unique alcohol use being even more strongly positive and significant, and life satisfaction and AUD being more strongly negative and significant.

## Methods

### Summary of cohorts

We obtained summary statistics for alcohol phenotypes and life satisfaction using existing large-scale datasets described below and summarized in Table 1. We only conducted analyses on those of European Ancestry due to the scarcity of large-scale summary statistics available within other ancestral groups.

**Table 1:** Descriptive information about phenotypes planned to be included.

Dataset	Phenotype	Accompanying GWAS	N	N effective
PGC-SUD	Alcohol dependence (AD) case/control	Walters et al. 2018	45,568 [N = 11,569 cases, N = 34,999 controls]	34,780
MVP	Alcohol use disorder (AUD) case/control	Kranzler et al. 2019	267,391 [N = 55,584 cases, N = 218,807 controls]	152,332
MVP	Max alcohol consumption	Gelernter et al. 2019b	126,936	126,936
UKB	AUDIT problems	Sanchez-Roige, Palmer et al. 2019	121,604	121,604
23andMe	AUDIT total*	Sanchez-Roige, Fontanillas et al. 2019	20,328	20,328
UKB	AUDIT consumption	Sanchez-Roige, Palmer et al. 2019	121,604	121,604
MVP	AUDIT consumption	Kranzler et al. 2019	206,254	206,254
GSCAN and UKB	Drinks per Week	Liu et al. 2019	941,280	941,280
UKBiobank	Satisfaction-family	Becker et al. 2021	168,313	168,313
UKBiobank	Satisfaction-finance	Becker et al. 2021	169,051	169,051
UKBiobank	Satisfaction-friends	Becker et al. 2021	168,001	168,001
UKBiobank	Satisfaction-work	Becker et al. 2021	115,038	115,038

*PGC-SUD* psychiatrics genomics consortium substance use disorder workgroup, *MVP* million veteran program, *UKB* United Kingdom biobank, *GSCAN* GWAS & sequencing consortium of alcohol and nicotine use.

*AUDIT-T from 23andMe not included in final models.

### Alcohol-related cohorts and phenotypes

#### AUD-related phenotypes

AUD case/control status came from the Million Veteran Program (MVP) dataset and was defined according to ICD-9 or ICD-10 codes for dependence or abuse diagnoses from Veteran’s Affairs electronic health records (EHR). Participants with at least one inpatient and/or two outpatient alcohol-related ICD-9/10 codes (from 2000 to 2018) were considered to be AUD cases^9^. AUD case/control status was available for 267,391 participants in MVP (N = 55,584 cases, N = 218,807 controls). Alcohol dependence case/control data came from a PGC-SUD meta-analysis^14^, which included over 20 datasets. Cases were defined to be meeting criteria for a DSM-IV (and DSM-III-R for one study) diagnosis of alcohol dependence and all controls were alcohol exposed (N = 46,568; N = 11,569 cases, N = 34,999 controls).

#### Alcohol use-related phenotypes

Drinks per week (DPW), defined as the average number of drinks a participant reported drinking each week, was examined in a combined approach with GSCAN consortium and UK Biobank (UKB)^17^ (N = 941,280); see Table 1. In studies that reported binned response ranges (e.g., 1–4 drinks), the midpoint of the range was utilized^17^. The AUDIT^29^ was available in multiple forms and studies. First, the AUDIT total score (AUDIT-T), was available in the 23andMe dataset^30^ for 20,328 participants. Second, data from the AUDIT- C subscale, which consists of three items measuring past-year typical quantity and frequency of drinking and frequency of heavy/binge drinking^31^, were available in two datasets: EHR data from the annual AUDIT-C assessment in MVP collected on individuals between 2007–2017 N = 206,254; Ref.^9^ and as part of the full 10-item AUDIT in the UK Biobank N = 121,604; Ref.^21^. Third, the AUDIT-Problems (P) subscale, consisting of 7 items that focus on the problematic consequences of drinking, was used from the UK Biobank (N = 121,604). Finally, in MVP, a quantitative measure of maximum habitual alcohol use in a typical month MaxAlc; Ref.^32^ was used to reflect typical/habitual maximum use (N = 126,936).

### Life satisfaction phenotypes

Summary statistics for the life satisfaction items were taken from the Social Science Genetic Association Consortium (SSGAC; https://www.thessgac.org/). Four items captured the extent to which individuals felt satisfied with their family, friends, work, and finances (e.g., “In general, how satisfied are you with the work that you do?”) with answer choices being 1–6 and higher scores indicating more satisfaction in each domain. These satisfaction items have been shown to be a good proxy for subjective well-being^33^. The summary statistics coming from genome-wide association studies for each of these four domains were used as indicators of the life satisfaction factor in the current analyses. Within the SSGAC website, satisfaction with family (n = 168,313), friends (n = 168,001), work (115,038), and finances (n = 169,051) were taken from UK Biobank^34^.

### Genotyping, quality control, and imputation

Summary statistics used in these analyses have undergone quality control pipelines applied by the specific consortia (e.g., PGC quality control pipeline including filtering to remove SNPs with imputation information value < 0.90 and minor allele frequency/MAF < 0; 01; Sullivan, 2010). The analytic pipeline for these analyses incorporates additional filtering keeping approximately 1,200,000 SNPs for each phenotype with the exception of the MVP analyses which keep approximately 625,000; see Ref.^25^ including removing variants that are not SNPs or are strand ambiguous, and removing SNPs based on a minimum N.

### Genomic structural equation modeling

We conducted analyses using the GenomicSEM package in R (version 0.0.3; https://github.com/GenomicSEM/GenomicSEM/wiki). GenomicSEM uses a two-stage SEM approach (Grotzinger et al., 2019). In the first stage, the covariance matrix and sampling covariance matrix are estimated for each dataset (see Supplementary Table 1). In the second stage, a SEM is specified and parameters are estimated by minimizing the discrepancy between the model-implied genetic covariance matrix and the empirical covariance matrix. The fit of the model can then be evaluated using standard metrics, including the standardized root mean square residual (SRMR), model chi square, Akaike Information Criterion (AIC), and the Comparative Fit Index (CFI)^35,36^.

Precomputed linkage disequilibrium (LD) scores were obtained from the 1000 Genomes Project, specifically the Europeans subsample (https://data.broadinstitute.org/alkesgroup/LDSCORE/eur_w_ld_chr.tar.bz2). For case/control samples, liability scale estimates assumed a population prevalence of 15.9% for alcohol dependence and AUD^14^.

### Data analytic plan

#### Factor analyses: overview of different factor models to be tested

Some of the text from this section is overlapping with our prior published gSEM manuscript^26^. The first thing we did was we asked whether a common factor model in which all alcohol-related and life satisfaction indicators loading on the same factor (Model A) would fit the data well. If Model A fits best, it would mean that these items are part of one underlying latent factor (versus more than one). Next, we tested whether either a two-factor model with all alcohol items loading on one factor and life satisfaction loading on a second factor (Model B) or a correlated three-factor model allowing for separate life satisfaction, alcohol use, and AUD-related factors (Model C) would provide better fit. If these models fit best it would mean that (Model B) the genetic influences on all alcohol indicators and life satisfaction are distinct, or that (Model C) the genetic influences on the alcohol use indicators are distinct from the AUD indicators and the life satisfaction indicators. We then asked whether a more complicated, Bifactor model that allows for factors common and specific to life satisfaction and alcohol phenotypes (Model D) would fit the data well, and whether the inter-factor correlations between life satisfaction and alcohol use and life satisfaction and AUD would differ from one another when estimating a factor common to all alcohol phenotypes. If this Model D fits best, it suggests that there are genetic influences that are common to all alcohol items, but that there are also importance genetic influences that are specific to alcohol use, and AUD, and also that the genetic influences on life satisfaction are distinct from the alcohol items. In Bifactor models, all items are allowed to load on one common factor and on their specific group factors. The group factors are allowed to correlate with one another, but their correlations with the general factor are usually set to zero. Typically, within Bifactor models, each item loads on the common factor and one specific factor. However, here the 23andMe AUDIT-T item was initially allowed to load on both alcohol use and AUD, as it is composed of items related to both use (AUDIT-C) and problems (AUDIT-P). We hypothesized that, within this framework, the genetic correlation between positive items and AUD would be significant and negative, the correlation between life satisfaction and alcohol use would be significant and positive, and the genetic correlation between life satisfaction and the common alcohol factor would be non-significant.

#### Factor analyses: determining number of factors

To determine which model best fit the data, we examined the substantive interpretability of each model and its loadings, including the genetic associations between the life satisfaction factor and factors common and specific to alcohol use and AUD. We also examined goodness-of-fit indices with the standard cut-offs for good fit, including a CFI: ≥ 0.9 and SRMR ≤ 0.08 and lower AIC values suggesting better fit and parsimony^37,38^. We used the zero-order genetic correlations between life satisfaction and alcohol phenotypes generated from this same author group^39^ to inform which alcohol items would load onto the alcohol use-related factor (i.e., drinks per week, AUDIT-C, and AUDIT-T) or the AUD-related factor (i.e., AUDIT-T, Max Alc, AUDIT-P, AUD, and alcohol dependence).

## Results

### Zero-order genetic correlations among life satisfaction items

In examining the genetic correlations among the four life satisfaction items, all were significantly correlated with one another (p < 0.001). The associations were all moderate to large (absolute value *r*_*g*_: 0.34–0.85). Thus, we proceeded with including all four items in a one factor model. This one factor model showed great fit to the data (χ: 54.78, AIC = 70.78, CFI = 0.97, SRMR = 0.08). All standardized loadings of items on this factor were stronger than + /− 0.4 (absolute value range: 0.50–0.88).

### Estimating initial models: a change to included items

Although the plan had been to include all items described above, upon viewing the loadings of the three factor model, the AUDIT-T from 23andMe had a near zero loading (−0.01, NS) on the AUD factor. Since the AUDIT-T is comprised of items capturing alcohol use (AUDIT-C) and alcohol problems (AUDIT-P), we opted to omit this item from the model entirely, rather than allowing it to load at a near zero level on one of those two factors and potentially water down the factor it loads on. Thus, the steps that are described in the next section pertain to the models we are calling “final”, in which all items described earlier—with the exception of AUDIT-T from 23andMe—were included.

### Estimating final models of alcohol and life satisfaction

#### Common factor model/model A

A single common factor model with all items except for AUDIT-T from 23andMe (Model A) did not fit the data well (χ^2^ = 3062.65, AIC = 3106.66, CFI = 0.60, SRMR = 0.22; Table 2) (Upon viewing the loadings of the three factor model, although all other loadings were stronger than + /− 0.4 (and p < .001), AUDIT-T from 23andMe had a near zero loading (-0.01, NS) on the AUD factor. Since the AUDIT-T is comprised of items capturing alcohol use and problems, we opted to omit this item from the model entirely. Thus, the steps that are described in this section pertain to the models we are calling “final”, in which all items described earlier—with the exception of AUDIT-T from 23andMe—were included). The loadings indicated that this factor was driven by the alcohol-related factors, while the loadings of the life satisfaction items were small and/or not statistically different from zero (Supplemental Table 2).

**Table 2:** Fit indices for Models.

Model	Χ^2^ *value (df)*	χ^2^ *p*-value	AIC	CFI	SRMR
A. Common factor	3062.66 (44)	p < 0.001	3106.66	0.60	0.22
B. Two correlated factors	882.33 (43)	p < 0.001	928.33	0.90	0.14
C. Three correlated factors	762.45 (41)	p < 0.001	812.45	0.90	0.11
D. Bifactor	553.54 (35)	p < 0.001	615.54	0.93	0.09

#### Two factor model/model B

The two-factor model (Model B) fit somewhat better, but not well (χ^2^ = 882.33, AIC = 928.33, CFI = 0.90, SRMR = 0.14; Table 2). For Model B, all the alcohol and life satisfaction loadings were significant (Supplemental Table 3). The genetic correlation between the life satisfaction factor and the alcohol factor was small and negative but significant (*r*_*g*_: − 0.07, p < 0.05).

#### Three factor model/model C

The three-factor model (Model C; Supplemental Table 4) fit adequately (χ^2^ = 762.45, AIC = 812.45, CFI = 0.90, SRMR = 0.11; Table 2). All of the loadings on these factors were significant. There was a small, negative association between AUD and the life satisfaction factor (*r*_*g*_: − 0.17, p < 0.001). The correlation between the alcohol use and life satisfaction was non-significant (*r*_*g*_: 0.00, NS), but the association between AUD and alcohol use was large, positive, and significant (*r*_*g*_: 0.72, p < 0.001).

#### Bifactor model/model D

**Figure 1:**
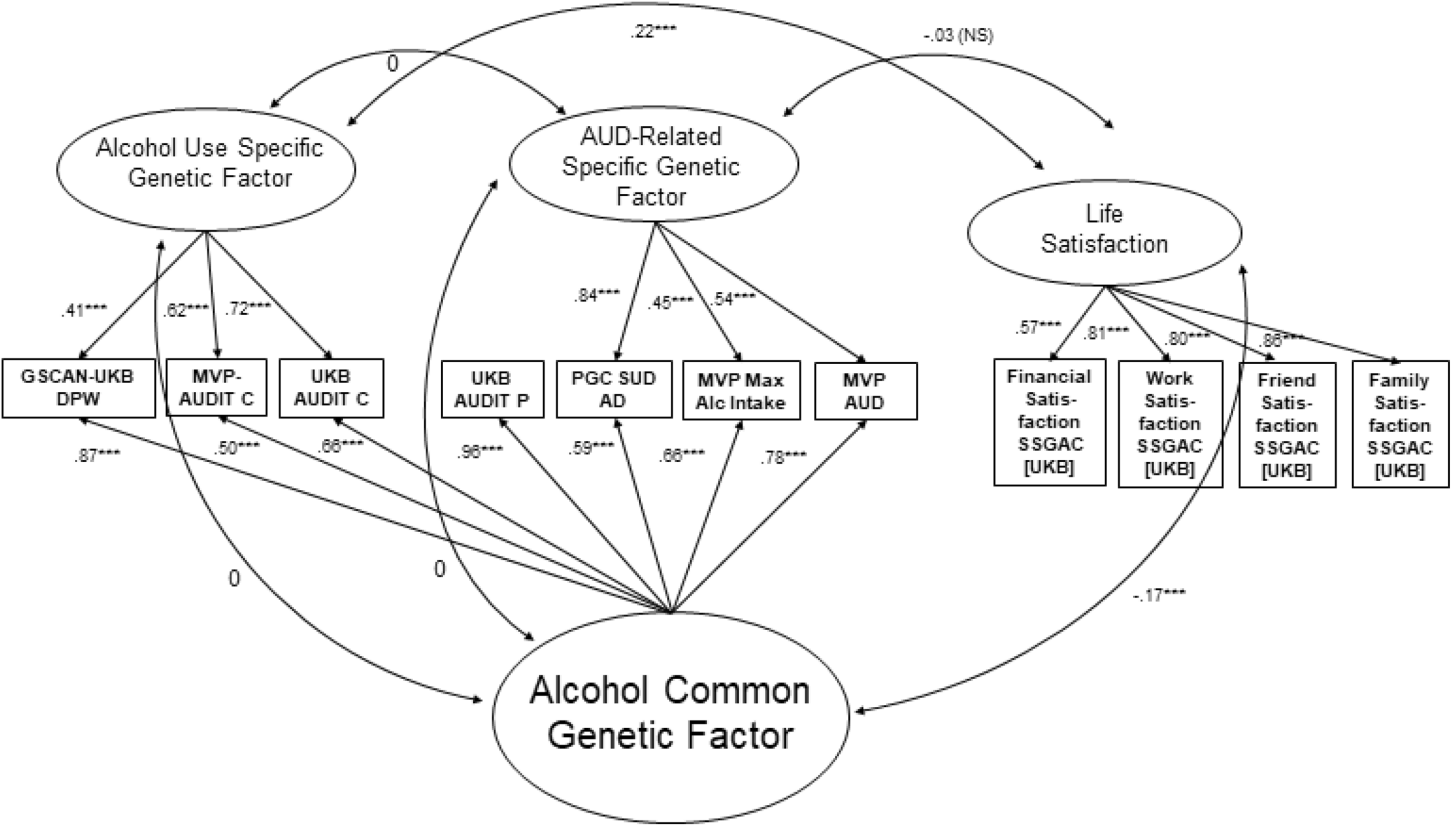
Depiction of Final, Bifactor Model. Standardized loadings and correlations are depicted; *p < 0.05, **p < 0.01, ***p < 0.001.

To test whether the associations between the life satisfaction factor, alcohol use, and AUD would shift when accounting for the common variation shared across all alcohol-related items, we fit a Bifactor model (Model D; See Fig. 1 and Supplemental Table 5) in which the alcohol items loaded onto 3 factors: a common factor, a residual alcohol use factor and a residual AUD factor. The correlations across these factors were fixed to zero. Model D also estimated the correlations between the life satisfaction factor and each of the three alcohol factors. Model D fit the data well (χ^2^ = 553.54, AIC = 615.54, CFI = 0.93, SRMR = 0.09; Table 2) and provided the best fit across the considered models. The genetic correlation between the life satisfaction factor and the common alcohol factor was negative (*r*_*g*_: − 0.17, p < 0.001). The correlation between the life satisfaction factor and the unique AUD factor was not significant (*r*_*g*_ − 0.03, NS). Finally, the correlation between the life satisfaction factor and the unique alcohol use factor was positive (*r*_*g*_: 0.22, p < 0.001).

## Discussion

We employed gSEM to examine multivariate associations between alcohol use, AUD, and life satisfaction. This study builds upon our previous work demonstrating both improved model fit after partitioning genetic variance into separate alcohol use and AUD factors^26^ and differential genetic associations across these factors and PTSD^22^. Based on this prior work, we sought to explore potential mechanisms that may contribute to these differences in the genetic architecture between PTSD and alcohol use versus PTSD and more problematic forms of alcohol use, such as AUD. Given the positive genetic correlations between PTSD and AUD compared to the negative or non-significant genetic correlations between PTSD and alcohol use^22^, we hypothesized that the alcohol use and AUD genetic factors may represent drinking patterns influenced by distinct reasons for drinking. As such, we hypothesized that genetic correlations between unique alcohol use and life satisfaction and unique AUD and life satisfaction would be in opposing directions (i.e., positive and negative, respectively).

### Best fitting model and its implications

A Bifactor model partialling out the shared variance across all alcohol-related items was the best fitting model. Therefore, associations between factors in that model are the focus of our interpretation and discussion. Consistent with our hypotheses, we found evidence of a positive genetic correlation between unique alcohol use and life satisfaction. This is consistent with phenotypic research indicating that alcohol use is positively associated with general well-being among low-risk (but not high-risk) drinkers^40^ and that low-risk drinkers report higher levels of general well-being compared to individuals who abstain from alcohol^41,42^. Previous research demonstrating that socially-motivated factors may mediate the relationship between happiness and alcohol use^43^ points to the possibility that individuals who drink alcohol at higher, but not necessarily problematic, levels tend to do so out of a desire to be social and that this may, in turn, be associated with higher general life satisfaction. Previous work demonstrating that social motives for drinking are the most commonly reported reasons for drinking^44,45^ and have been shown to be positively associated with frequency and quantity of use, but not to heavy drinking or alcohol-related problems^46^ may lend further support for this theory. Another possible explanation for the positive genetic association between alcohol use and life satisfaction could be the “healthy volunteer” effect, such that large population-based cohorts like those used in the current gSEM analyses tend to be overall healthier and of higher socioeconomic status than the broader population. As such, it is possible that our results are subject to selection bias. This aligns with previous work demonstrating that individuals of higher socioeconomic status tend to endorse more frequent alcohol use in the absence of non-problematic alcohol use^18,47^.

### Revisiting hypotheses about correlations among factors

Our hypothesis that the genetic association between unique AUD and life satisfaction would be negative was not supported, as we did not find evidence for any association between the two genetic factors. Our hypothesis was formulated based on prior work demonstrating positive genetic associations between AUD/AD and PTSD in across both behavioral genetic^48^ and molecular genetic^22,26,49^ research designs. We had thought that the AUD factor might represent the propensity to drink for reasons more consistent with negatively valenced intentions, such as to avoid aversive emotional states, specifically in the context of PTSD^50^. However, prior work suggests that psychopathology and life satisfaction are distinct, albeit related, phenotypes^51–53^ and, therefore, it is possible our findings reflect a similar genotypic distinction. The null association between life satisfaction and the alcohol factor unique to disordered use suggests that genetic risk for problematic/pathological alcohol use is not associated with genetic risk for life dissatisfaction. This would be consistent with some phenotypic work suggesting that psychopathology and psychological well-being (e.g., life satisfaction, self-realization, social well-being) are not two ends of a single dimension^52,53^.

We also tested the genetic association between the common factor reflecting shared variance across alcohol use and AUD with life satisfaction. Contrary to our hypothesis that this common factor would show no significant association with life satisfaction as was the case with PTSD in prior work; Ref.^26^, the common factor was negatively associated with life satisfaction in the present study. One possible explanation for this negative association could be that the genetic signal that is shared across all alcohol use indicators, including measures of alcohol use in the general population, is generally indexing genetic propensities for heavier drinking. This may then indicate patterns of drinking associated with, or motivated by, overall life dissatisfaction. Conversely, it is possible that drinking at high levels leads to greater life dissatisfaction across the domains captured by the indicators of the life satisfaction factor in the present study (e.g., work, family, friends, finances). There is phenotypic support for the bidirectional nature of these two constructs. Specifically, findings from a large (*n* = 14,083), 15-year longitudinal study of healthy Finnish twins^54^ showed that life dissatisfaction and adverse alcohol use (including binge drinking, passing out, high use) reciprocally influenced each other over time, and that the magnitude of this relationship increased with heavier alcohol use.

The lack of evidence of a genetic association between AUD and life satisfaction, as well as the negative genetic association between the common alcohol-related factor and life satisfaction were unexpected in light of previous findings presented by our group. Specifically, using gSEM techniques, we reported that AUD is negatively genetically correlated with PTSD and that there was no evidence of a genetic association between PTSD and the common factor representing shared genetic variance across AUD and use^26^. The discrepancy between these findings and those from the present study highlight a few important points. First, in our prior work, the PTSD factor was capturing what is common between PTSD case/control status and PTSD Re-experiencing symptoms (i.e., the two indicators); that is, the factor is primarily measuring intrusive symptoms such as nightmares, flashbacks, and repetitive, distressing images, typically related to the experienced traumatic event. In contrast, our life satisfaction factor was capturing what is common among indicators of family, friend, work, and financial satisfaction—so some general contentment with all these areas of life. In phenotypic analyses, more intrusive trauma-related (PTSD) symptoms tend to be associated with thought disorders/conditions such as mania psychosis^55^, while higher satisfaction tends to be associated with more happiness, well-being, and less neuroticism, loneliness, and depressive symptoms^33^. Second, and relatedly, it seems important to apply gSEM to questions involving positive constructs, such as life satisfaction and other positively valenced constructs associated with psychological well-being, in addition to psychiatric disorders. Doing so not only provides biological support for the notion that life satisfaction and psychopathology are not opposite ends of the same dimension, but also allows for the identification of novel patterns between previously studied phenotypes and other constructs relevant to, but distinct from, psychopathological functioning. As such, incorporation of positively valenced constructs into gSEM provides clinically relevant insight into the potential biological impact of specific treatment intervention and prevention approaches (e.g., interventions to increase life satisfaction).

### Limitations

The present findings should be interpreted in light of several limitations. First, the summary statistics that were available for our multiple phenotypes of interest, particularly when collated across various datasets, were limited to individuals of European Ancestry. This is problematic for a number of reasons, including the fact that initial work from our group demonstrated that zero order genetic correlations between PTSD and alcohol-related constructs differed across European Ancestry and admixed populations^22^. Exploring these associations among more diverse populations will become more feasible as more summary statistics including individuals from other ancestral groups become available. Additionally, while not inclusive of all individuals/populations, particularly outside of European Ancestry, the datasets from which gSEM analyses were conducted in general are large and representative, or are from consortia of numerous studies, adding to the generalizability of the findings. Second, the available summary statistics for alcohol-related phenotypes precluded analyses split by sex, which is problematic given known sex differences in molecular genetic associations between alcohol-related phenotypes and other constructs, such as PTSD^49^, as well as known sex differences in the prevalence of alcohol-related phenotypes^56^, motives for drinking^46^, and relationships between life satisfaction and alcohol use^57^. Third, due to modeling issues, the 23andMe AUDIT-T item was excluded from each of the models. This choice was made in large part to statistical reasons; however, for substantive reasons as well, it makes sense that this item was not included. It was important for us to examine the genetic architecture of alcohol use and AUD-related phenotypes separately, and the AUDIT-T scale included both consumption and problems items. Thus, it does not make clear sense on which factor(s) that item would load. Additionally, due to other modeling issues, the PGC AD factor was excluded from the unique AUD factor. These modifications resulted in a Bifactor model that differed from that which was run in our earlier paper^26^. Finally, these findings should be interpreted in the context of the authors not having pre-registered study hypotheses.

### Take-home points and implications

Despite these limitations, this study advances the field by applying a novel methodology (i.e., gSEM) to a question relating to a positive psychological construct, which is rare in the field of psychiatric genetics, and is of high importance, given the potential to increase our understanding surrounding the etiology of alcohol phenotypes and prevention of problematic alcohol use. This study also adds to a growing body of work suggesting that the genetic architecture of alcohol use and AUD are distinct and that these distinctions extend to external correlates ranging from psychopathology (e.g., PTSD) and psychological well-being (e.g., life satisfaction). This work also provides some initial evidence for shared etiology across life satisfaction and typical alcohol use, and life dissatisfaction and heavy alcohol use. Although our findings reflect association and not causality, they provide some foundation on which longitudinal, causal models might build. Specifically, work testing whether intervening on life dissatisfaction reduces heavy alcohol use specifically, may be a beneficial next step in this line of research.

### Supplementary Information

Supplementary Tables.

## Data Availability

All data analyzed are summary-level data, most of which are publicly available to qualified investigators (e.g., https://pgc.unc.edu/for-researchers/download-results/). The exception to this is summary-level data coming from the Million Veterans Program (MVP), which is available with a data use agreement through dbGaP.
