# Supplementary material for: Genetic associations between alcohol phenotypes and life satisfaction: a genomic structural equation modelling approach
Source: Sci Rep. 2023 Aug 18;13:13443. doi: 10.1038/s41598-023-40199-1 (PMC10439217; doi:10.1038/s41598-023-40199-1)
Supplement: Supplementary file 1 — Supplementary Tables. [file 41598_2023_40199_MOESM1_ESM.docx]

*Supplemental Table 1.* Zero-order Genetic Correlations between Phenotypes.

|  | 1. | 2. | 3. | 4. | 5. | 6. | 7. | 8. | 9. | 10. | 11. |
| --- | --- | --- | --- | --- | --- | --- | --- | --- | --- | --- | --- |
| 1. PGC AD | 1 |  |  |  |  |  |  |  |  |  |  |
| 2. MVP AUD | .98*** | 1 |  |  |  |  |  |  |  |  |  |
| 3. UKB AUDIT-C | .28** | .48*** | 1 |  |  |  |  |  |  |  |  |
| 4. UKB AUDIT-P | .60*** | .71*** | .69*** | 1 |  |  |  |  |  |  |  |
| 5. MVP Max Alcoholic Drinks | .84*** | .76*** | .41*** | .69*** | 1 |  |  |  |  |  |  |
| 6. MVP AUDIT-C | .16 | .47*** | .78*** | .49*** | .23*** | 1 |  |  |  |  |  |
| 7. 23andMe AUDIT-T(*) | .16 | .58*** | .73*** | .58*** | .52*** | .72*** | 1 |  |  |  |  |
| 8. GSCAN and UKB Drinks per Week | .65*** | .68*** | .85*** | .81*** | .61*** | .72*** | .77*** | 1 |  |  |  |
| 9. Financial Satisfaction | -.53*** | -.25*** | .06 | -.20** | -.27*** | .20*** | .33*** | -.06 | 1 |  |  |
| 10. Work Satisfaction | -.14 | -.10 | .02 | -.05 | .01 | -.01 | .10 | .05 | .59*** | 1 |  |
| 11. Friend Satisfaction | .07 | -.02 | .02 | -.17** | .05 | -.01 | -.07 | -.02 | .34*** | 67*** | 1 |
| 12. Family Satisfaction | -.19* | -.09* | .06 | -.16** | -.06 | -.02 | .04 | -.06 | .39*** | .63*** | .85*** |

*Note.* **p*<.05, ***p<*.01*, ***p<.*001. (*)AUDIT-T from 23andMe not included in final models.

*Supplemental Table 2*. Parameter estimates for Common Factor Model (Model A).

|  | **Standardized Estimate (loading)** | **Standard Error** |
| --- | --- | --- |
| **Alcohol and Positive Common Factor** |  |  |
| PGC-AD | .67*** | .05 |
| MVP-AUD | .79*** | .03 |
| UKB AUDIT-C | .77*** | .03 |
| UKB AUDIT-P | .87*** | .03 |
| MVP-Max Alcoholic Drinks | .70*** | .04 |
| MVP-AUDIT-C | .64*** | .03 |
| GSCAN and UKB-Drinks Per Week | .93*** | .03 |
| Financial Satisfaction | -.14*** | .03 |
| Work Satisfaction | -.05 | .04 |
| Friend Satisfaction | -.05 | .03 |
| Family Satisfaction | -.10** | .03 |

Note: *p<.05, **p<.01, ***p<.001.

*Supplemental Table 3*. Parameter estimates for Two Factor Model (Model B).

|  | **Standardized Estimate (loading or correlation)** | **Standard Error** |
| --- | --- | --- |
| **Alcohol Factor** |  |  |
| PGC-AD | .66*** | .05 |
| MVP-AUD | .78*** | .03 |
| UKB AUDIT-C | .78*** | .03 |
| UKB AUDIT-P | .87*** | .03 |
| MVP-Max Alcoholic Drinks | .69*** | .04 |
| MVP-AUDIT-C | .65*** | .03 |
| GSCAN and UKB-Drinks Per Week | .94*** | .03 |
| **Life Satisfaction Factor** |  |  |
| Financial Satisfaction | .52*** | .04 |
| Work Satisfaction | .81*** | .05 |
| Friend Satisfaction | .84*** | .04 |
| Family Satisfaction | .88*** | .04 |
| **Correlation between Factors** |  |  |
| Alcohol-Life Satisfaction Items | -.07 | .03 |

Note: *p<.05, **p<.01, ***p<.001.

*Supplemental Table 4.* Parameter estimates for Three-Factor Model (Model C).

|  | **Standardized Estimate (loading or correlation)** | **Standard Error** |
| --- | --- | --- |
| **Alcohol Use Disorder (AUD) Specific Factor** |  |  |
| PGC-AD | .76*** | .06 |
| MVP-AUD | .89*** | .04 |
| UKB AUDIT-P | .94*** | .03 |
| MVP-Max Alcoholic Drinks | .77*** | .04 |
| **Alcohol Use Specific Factor** |  |  |
| UKB AUDIT-C | .84*** | .03 |
| MVP-AUDIT-C | .69*** | .04 |
| GSCAN and UKB-Drinks Per Week | 1.05*** | .03 |
| **Positive Items Factor** |  |  |
| Financial Satisfaction | .56*** | .04 |
| Work Satisfaction | .82*** | .05 |
| Friend Satisfaction | .81*** | .04 |
| Family Satisfaction | .86*** | .04 |
| **Correlations between factors** |  |  |
| AUD- Life Satisfaction Items | -.17*** | .03 |
| Alcohol Use- Life Satisfaction Items | .00*** | .03 |
| AUD-Alcohol Use | .72 | .02 |

Note: *p<.05, **p<.01, ***p<.001.

*Supplemental Table 5*. Parameter estimates for Bifactor Model (Model D).

|  | **Standardized Estimate (loading or correlation)** | **Standard Error** |
| --- | --- | --- |
| **Alcohol Use Disorder (AUD) Specific Factor** |  |  |
| PGC-AD | .84*** | .17 |
| MVP-AUD | .54*** | .09 |
| MVP-Max Alcoholic Drinks | .45*** | .08 |
| **Alcohol Use Specific Factor** |  |  |
| UKB AUDIT-C | .72*** | .08 |
| MVP-AUDIT-C | .62*** | .08 |
| GSCAN and UKB-Drinks Per Week | .41*** | .04 |
| **Life Satisfaction Factor** |  |  |
| Financial Satisfaction | .57*** | .04 |
| Work Satisfaction | .81*** | .05 |
| Friend Satisfaction | .80*** | .04 |
| Family Satisfaction | .86*** | .04 |
| **Common Alcohol Factor** |  |  |
| PGC-AD | .59*** | .06 |
| MVP-AUD | .78*** | .04 |
| UKB AUDIT-P | .96*** | .03 |
| MVP-Max Alcoholic Drinks | .66*** | .05 |
| UKB AUDIT-C | .66*** | .03 |
| MVP-AUDIT-C | .50*** | .04 |
| GSCAN and UKB-Drinks Per Week | .87*** | .04 |
| **Correlations between factors** |  |  |
| AUD- Life Satisfaction Items | -.03 | .08 |
| Alcohol Use- Life Satisfaction Items | .22*** | .05 |
| Overall- Life Satisfaction Items | -.17*** | .04 |

Notes: *p<.05, **p<.01, ***p<.001. Correlations between AUD-alcohol use, AUD-Overall, and Alcohol Use-Overall are not displayed because they were all fixed to 0
